# Supplementary material for: Polymer nanoparticles pass the plant interface
Source: Nat Commun. 2022 Nov 30;13:7385. doi: 10.1038/s41467-022-35066-y (PMC9712430; doi:10.1038/s41467-022-35066-y)
Supplement: Supplementary file 1 — Supplementary Information [file 41467_2022_35066_MOESM1_ESM.pdf]

Supplementary information for Polymer nanoparticles pass the plant interface

Sam J. Parkinson, Sireethorn Tungsirisurp, Chitra Joshi, Bethany L. Richmond, Miriam L. Gifford, Amrita Sikder, Iseult Lynch, Rachel K. O'Reilly and Richard M. Napier

Supplementary Table *1*. Characterisation data obtained for polymer nanoparticles incubated with *Arabidopsis thaliana* roots

Polymer	M_n / g mol^{-1}	Đ (GPC)	D_h / nm	Đ (DLS)	ζ / mV
**$PDMAm_{70}$-$PDAAm_{50}$**	36,500	1.14	23	0.06	-3.1±5
**$PDMAm_{70}$-$PDAAm_{100}$**	48,200	1.14	37	0.02	-0.4±5
**$PDMAm_{70}$-$PDAAm_{200}$**	61,500	1.15	83	0.08	0.4±4
**$P(DMAm_{70,90\%}+AA_{70,10\%})$-$PDAAm_{50}$**	36,800	1.17	22	0.14	-1.9±3
**$P(DMAm_{70,90\%}+AA_{70,10\%})$-$PDAAm_{100}$**	51,100	1.15	50	0.09	-1.9±6
**$P(DMAm_{70,90\%}+AA_{70,10\%})$--$PDAAm_{200}$**	57,500	1.22	98	0.07	-2.0±7
**$PQDMAEMA_{70}$-$PDAAm_{100}$**	N/A	N/A	28	0.13	30.8±4
**$PQDMAEMA_{70}$-$PDAAm_{200}$**	N/A	N/A	40	0.09	23.8±2
**$PDMAPS_{70}$-$PDAAm_{50}$**	N/A	N/A	30	0.08	
**$P(DMAm_{70,75\%}+QDMAEMA_{70,25\%})$-$PDAAm_{100}$**	N/A	N/A	42	0.05	19.3±1
**$P(DMAm_{70,50\%}+QDMAEMA_{70,50\%})$-$PDAAm_{100}$**	N/A	N/A	40	0.08	27.2±1
**$P(DMAm_{70,25\%}+QDMAEMA_{70,75\%})$-$PDAAm_{100}$**	N/A	N/A	38	0.08	26.8±1
**$P(DMAm_{70,95\%}+AA_{70,5\%})$-$PDAAm_{50}$**	38,400	1.17	34	0.03	-3.4±5

Supplementary Table 2: Table of primers.

Gene	Forward primer	Reverse primer
**UBQ10, AT4G05320**	GGTTTGTGTTTTGGGGCCTTG	CGAAGCGATGATAAAGAAGAAGTTCC
**TIP41, AT4G34270**	GAACTGGCTGACAATGGAGTGT	GTTGGTGCCTCATCTTCGGG
**FRK1, AT2G19190**	ATCTTCGCTTGGAGCTTCTC	TGCAGCGCAAGGACTAGAG
**PHI1, AT2G21870**	TTGGTTTAGACGGGATGGTG	ACTCCAGTACAAGCCGATCC
**NHL10, AT2G35980**	TTCCTGTCCGTAACCCAAAC	CCCTCGTAGTAGGCATGAGC
**WRKY1, AT2G04880**	GGCAGCGTCTCCAATGGAAAA	TGCACTTATCGCCGGTACTCT

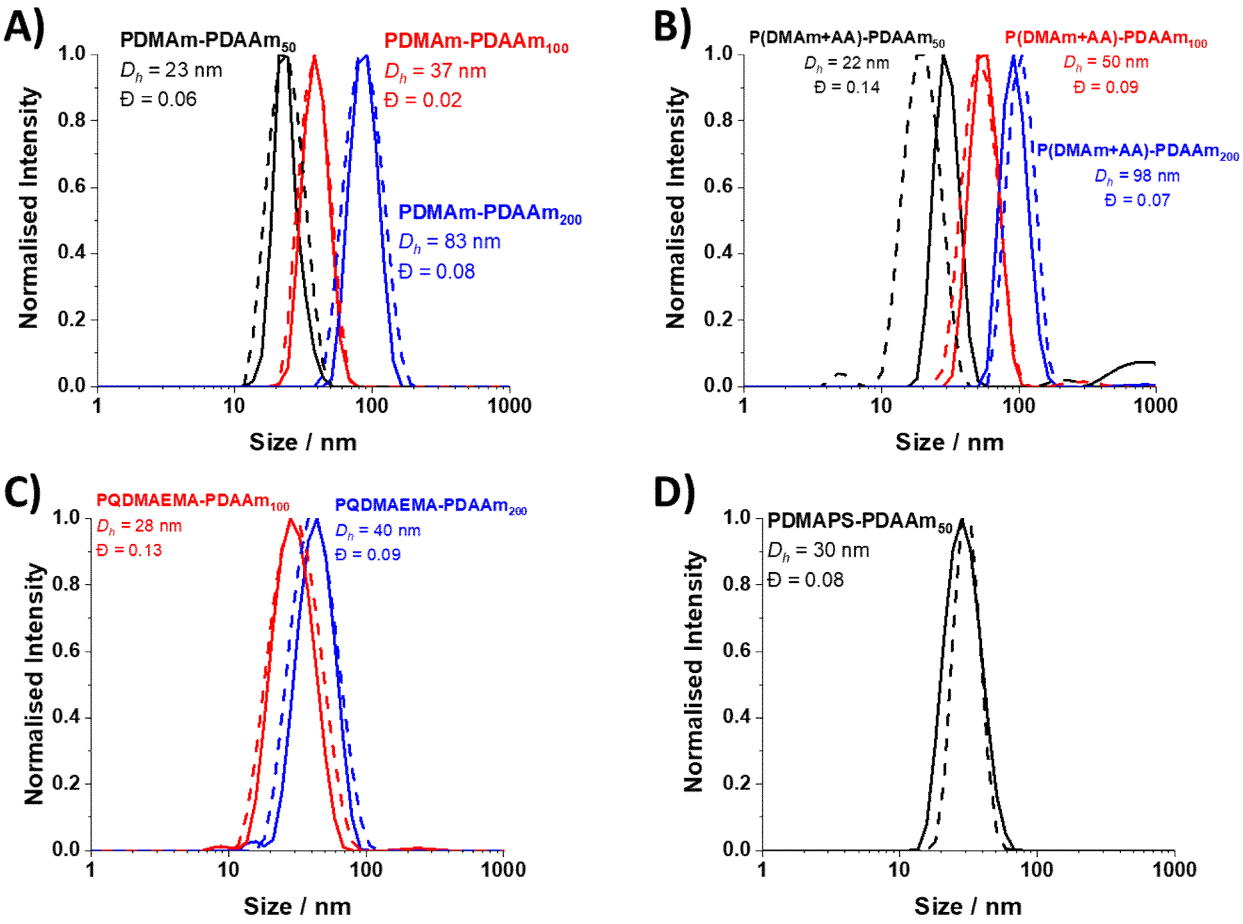

Supplementary Figure 1. DLS traces for a) PDMAm-PDAAm, b) P(DMAm+AA)-PDAAm, c) PQDMAEMA-PDAAm and d) PDMAPS-PDAAm nanoparticles both before (dashed) and after (solid) BODIPY attachment.

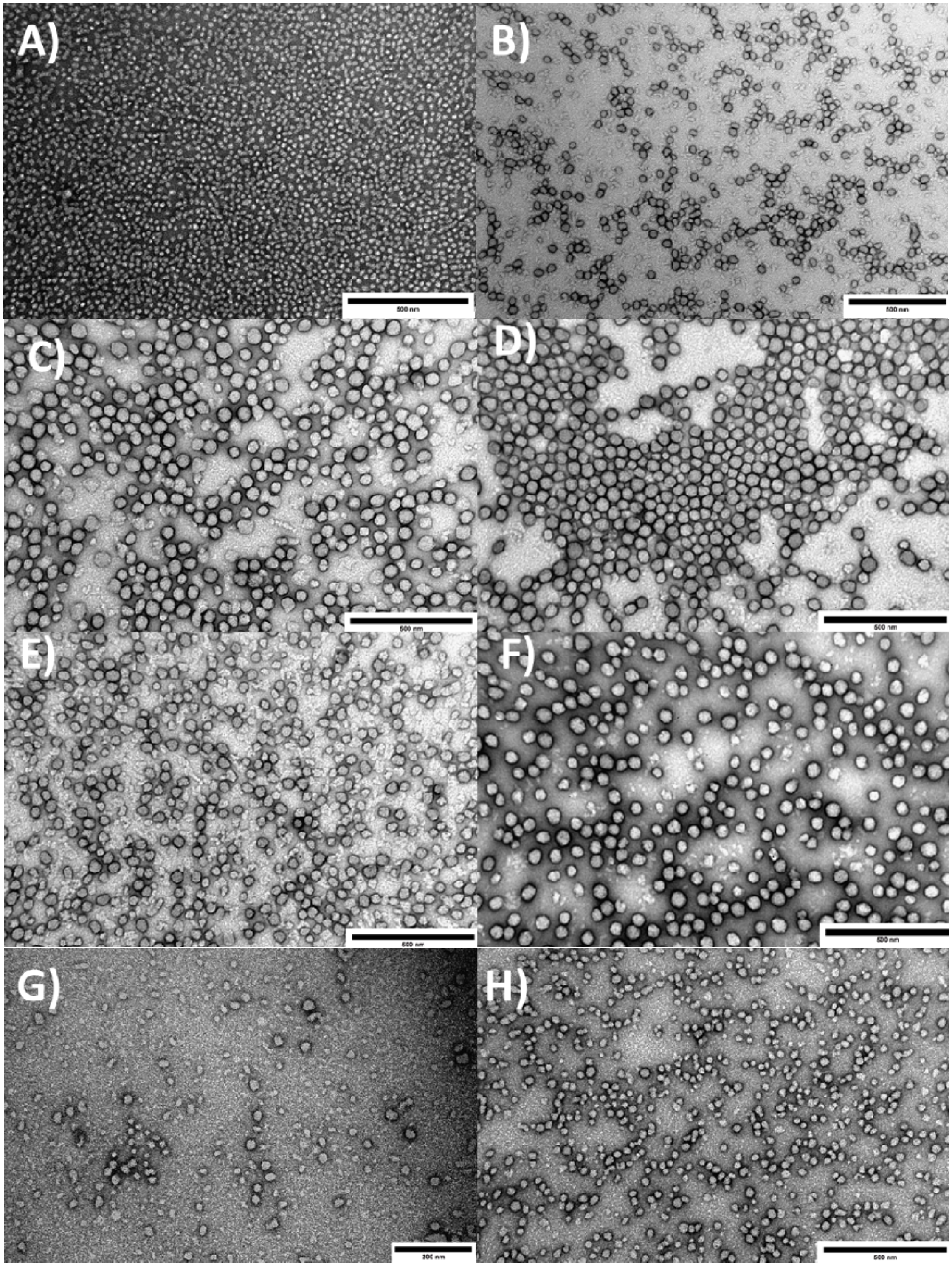

Supplementary Figure 2. Representative transmission electron microscopy images for a) PDMAm-PDAAm$_{50}$, b) PDMAm-PDAAm$_{100}$, c) PDMAm-PDAAm$_{200}$, d) P(DMAm+AA)-PDAAm$_{50}$, e) P(DMAm+AA)-PDAAm$_{100}$, f) P(DMAm+AA)-PDAAm$_{200}$, g) PQDMAEMA-PDAAm$_{100}$, h) PQDMAEMA-PDAAm$_{200}$. Scale bars differ and are given in each image.

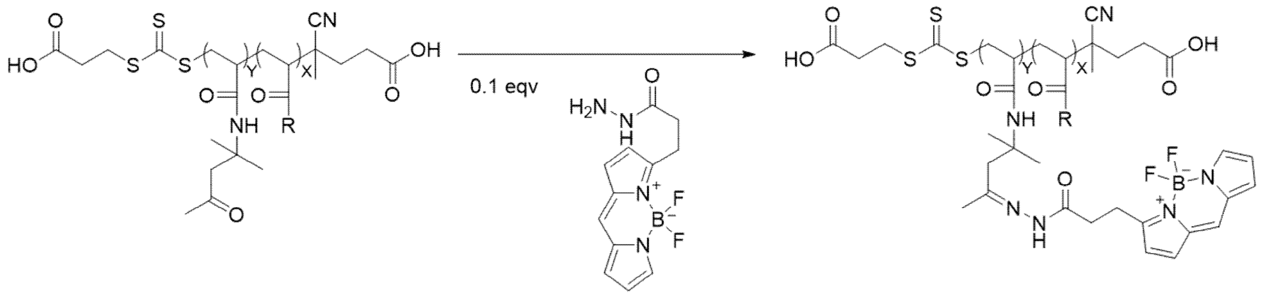

Supplementary Figure 3. Reaction scheme for BODIPY fluorophore attachment to polymeric nanoparticles.

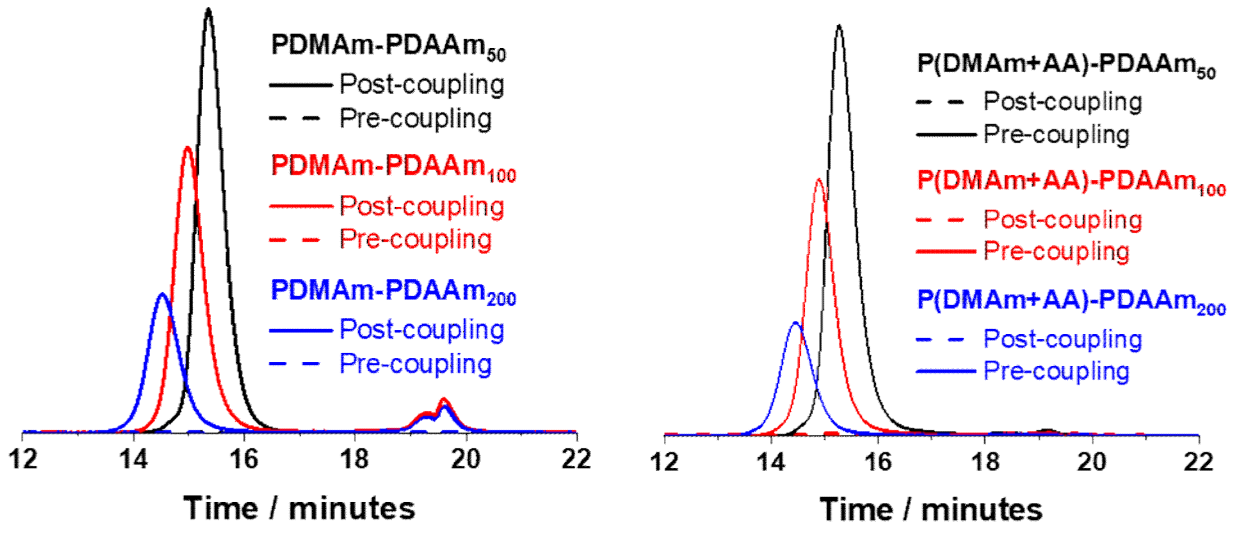

Supplementary Figure 4. UV-SEC traces obtained at 490 nm for a) PDMAm-PDAAm and b) P(DMAm+AA)-PDAAm nanoparticles before (dashed) and after (solid) BODIPY fluorophore attachment.

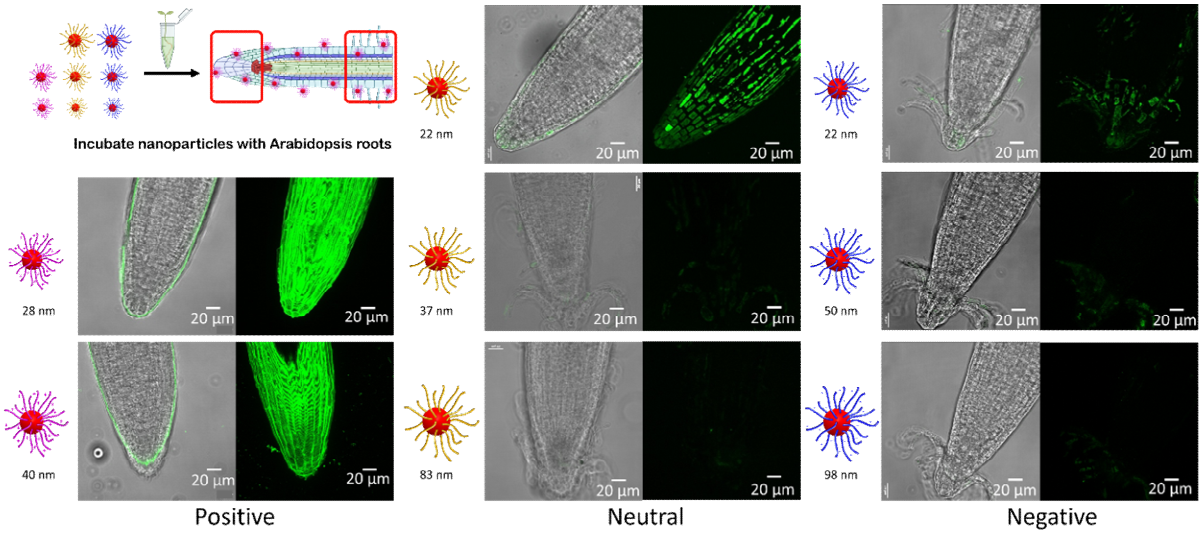

Supplementary Figure 5. Confocal images for the penetration (entry into cells) and accumulation (extent of nanoparticle build up in cells) of polymeric nanoparticles by *Arabidopsis* root tips. Penetration and accumulation were evaluated using a ZEISS 880 LSM. Maximum Z projections in the 488 nm laser channel were analysed alongside the Z-slices and merged with brightfield images using ImageJ software. Scale bar = 20 µm. The images are representatives of experimental replicates (n=3). Part of this figure was created with Biorender.com.

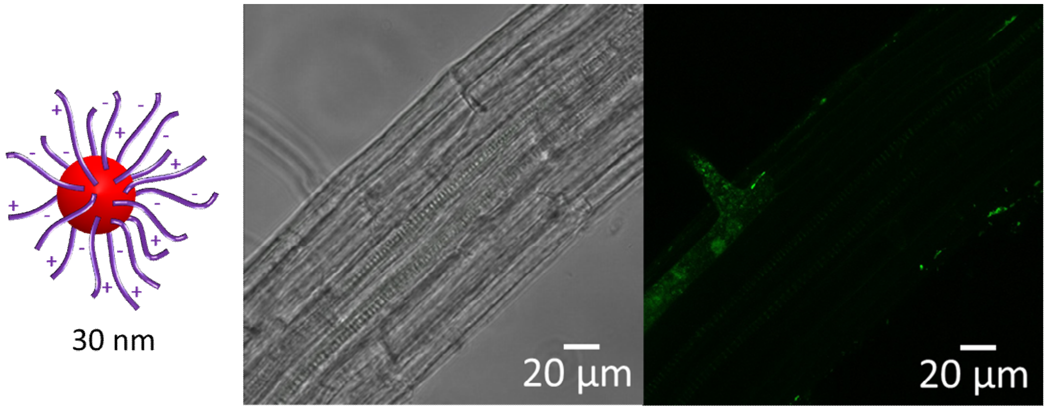

Supplementary Figure 6. Confocal images for the penetration and accumulation of zwitterionic polymeric nanoparticles in *Arabidopsis* root hair zones. Penetration and accumulation were evaluated under ZEISS 880 LSM. Maximum Z projections in 488 nm laser channel were analysed alongside the Z-slices and merged with brightfield images using ImageJ software. Scale bar = 20 µm. The images are representatives of experimental replicates (n=3).

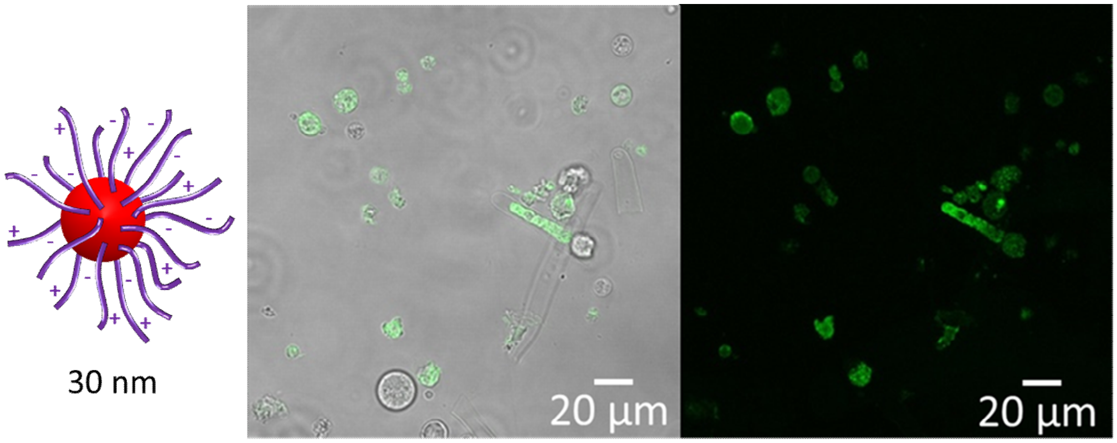

Supplementary Figure 7. Confocal images for the penetration and accumulation of zwitterionic polymeric nanoparticles in *Arabidopsis* protoplasts. Penetration and accumulation were evaluated under ZEISS 880 LSM. Maximum Z projections in 488 nm laser channel were analysed alongside the Z-slices and merged with brightfield images using ImageJ software. Scale bar = 20 µm. The images are representatives of experimental replicates (n=3).

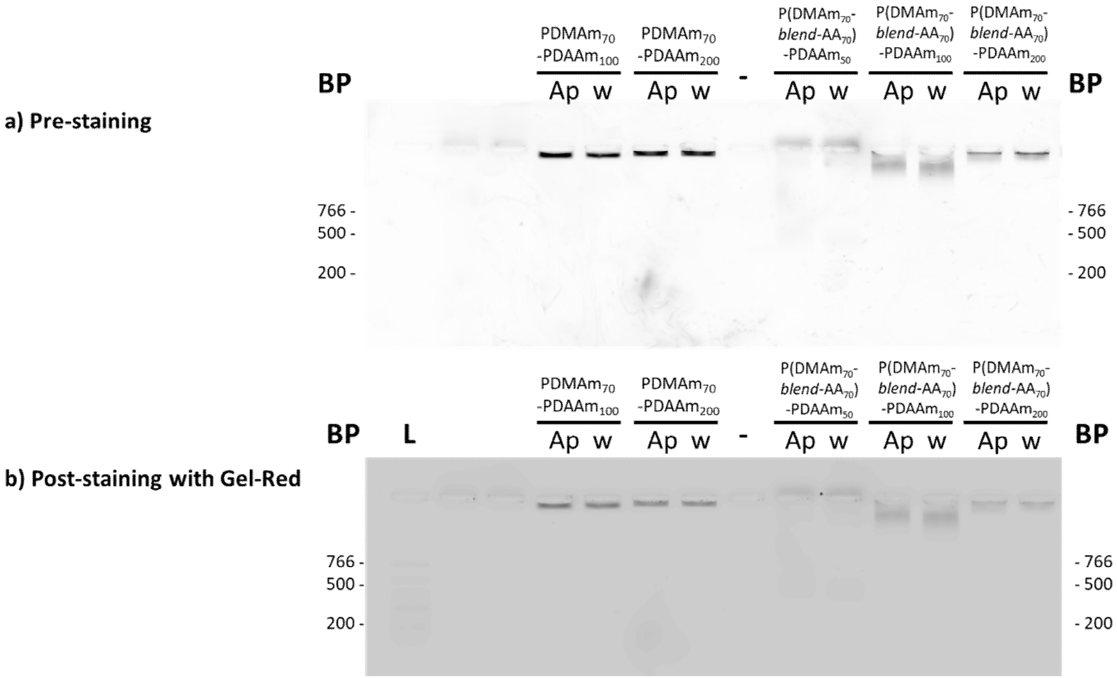

Supplementary Figure 8. Comparable stability of CPNs upon incubation in extracted apoplastic fluid from *Nicotiana benthamiana* leaves (Ap) in comparison to those incubated in distilled water (w). 2% agarose gel electrophoresis was performed with low M_w DNA ladder (L; Low Molecular Weight DNA Ladder, NEB UK #N3233) as a size standard. A negative control of apoplastic fluid alone (-) confirmed no background bands. Incubations were for 2.5 hours at room temperature. The gel was imaged using a Typhoon fluorescence imager with filters for BODIPY (top panel) before it was stained with Gel-Red and imaged again using the RGB filter (lower panel). No bleeding of the BODIPY away from the nanoparticle bands was observed. No quantitative scan of the gel was made.

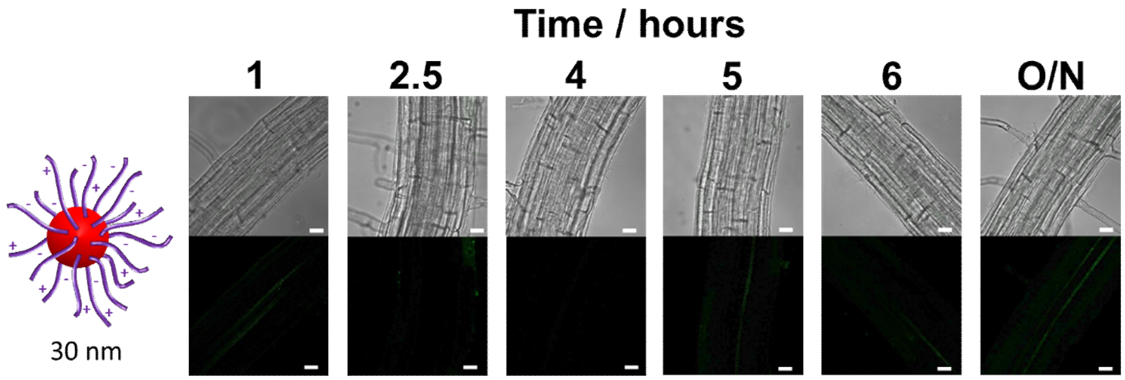

Supplementary Figure 9. Confocal images for the penetration and accumulation of zwitterionic polymeric nanoparticles in *Arabidopsis* root hair zones over time. Penetration and accumulation were evaluated under ZEISS 880 LSM. Maximum Z projections in 488 nm laser channel were analysed alongside the Z-slices and merged with brightfield images using ImageJ software. Scale bar = 20 µm. The images are representatives of experimental replicates (n=3).

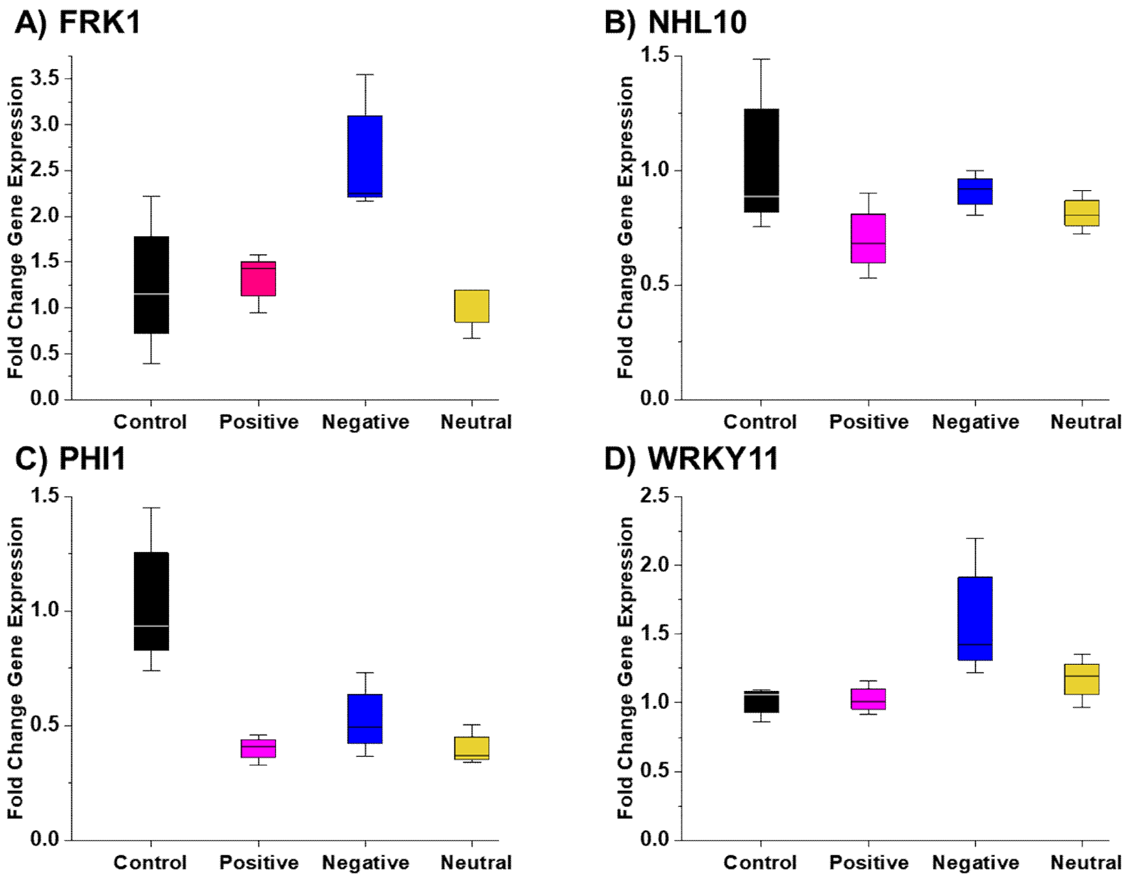

Supplementary Figure 10. Arabidopsis seedlings respond to treatment of their roots with nanoparticles by changes in gene expression. Four genes known to be associated with responses to stresses (Supplementary Table 3) have been used to monitor for rapid responses in gene expression in response to the nanoparticle treatments. Box plots showing fold changes with standard errors of the means represent data from three biological and three technical replicates of each sample. The centre lines denote the median value or 50th percentile and the boxes contain the 25th to 75th percentiles. The tip of the whiskers mark the minima (bottom) and maxima (top). The nanoparticle treatments were with the smallest particles in each case (see Figure 2), as used for the confocal microscopy (Figures 2-5). Nanoparticle treatments (using water as untreated control) were for 90 minutes. The data shown use TIP41 as reference gene. The only statistically significant change was seen with PHI1, which was down regulated in response to all the nanoparticles.

Supplementary Table 3. Stress genes and control, housekeeping genes selected to test for rapid responses to nanoparticles

Gene name	TAIR	GO Function annotation
**WRKY11**	AT4G31550	Defense response to bacterium
**FRK1**	AT2G19190	Encodes a receptor-like protein kinase that is involved in early defense signaling. Expression of this gene is strongly induced during leaf senescence.
**PHI1**	AT1G35140	Response to hypoxia
**NHL10**	AT2G35980	Defense response to virus, leaf senescence, response to other organism
